# Examination of viraemia and clinical signs after challenge with a heterologous PRRSV strain in PRRS Type 2 MLV vaccinated pigs: A challenge-dose study

**DOI:** 10.1371/journal.pone.0209784

**Published:** 2018-12-28

**Authors:** Greg Haiwick, Joseph Hermann, Michael Roof, Brian Fergen, Reid Philips, Abby Patterson

**Affiliations:** 1 Boehringer Ingelheim Animal Health Research and Development, Ames, Iowa, United States of America; 2 Boehringer Ingelheim Animal Health, Duluth, Georgia, United States of America; Plum Island Animal Disease Center, UNITED STATES

## Abstract

Vaccination with porcine reproductive and respiratory syndrome (PRRS) Type 2 modified-live vaccines (MLVs) has been shown to improve clinical signs and survival rates in PRRS virus (PRRSV)-challenged pigs. This study evaluated the dose of PRRSV challenge needed to cause and maintain viraemia in PRRS Type 2 MLV-vaccinated pigs and assessed clinical responses to various doses of virulent challenge. This controlled, randomised, blinded vaccination-challenge study involved 95 pigs who were either vaccinated with 2 mL of a PRRS Type 2 MLV on Day 0 or left unvaccinated. On Day 28, pigs were challenged intranasally with virulent PRRSV isolate (dose range <1.5 to 4 log_10_ 50% tissue culture infectious dose/mL). Five pigs were left unchallenged and served as environmental controls. Viraemia levels, pyrexia, average daily weight gain and clinical signs were assessed. At all challenge doses, vaccinated groups had reduced viraemia levels and clinical signs, and higher average daily weight gain compared with non-vaccinated groups. Vaccinated groups challenged with ≤2 log had similar viraemia levels and clinical performance (days pyrexic and average daily weight gain) as the non-challenged group. Vaccinated groups had significantly reduced pyrexic days compared with non-vaccinated groups across all challenge doses (P <.05). Vaccinated pigs challenged with <3 log had significantly improved average daily weight gain (P <.05). In vaccinated groups, challenge dose correlated positively with viraemia levels and number of days pyrexic, and negatively with average daily weight gain. This is the first study to use a challenge-dose model to evaluate the efficacy of vaccination against PRRSV. PRRS Type 2 MLV was shown to mitigate the consequences of PRRSV infection at all evaluated PRRSV challenge doses. Lower levels of challenge had minimal impact on health and performance of vaccinated pigs, supporting the benefit of vaccinating swine with PRRS Type 2 MLV.

## Introduction

Porcine reproductive and respiratory syndrome (PRRS) is responsible for substantial animal and economic losses to the swine industry [1]. The PRRS virus (PRRSV) causes viraemia, which typically leads to pyrexia, pneumonia with abnormal respiratory behaviour, and reduced average daily weight gain (ADWG) [2]. Previous studies have shown that pigs can be infected by low doses of the virus [3–5]. PRRSV is highly infectious and spreads via intranasal, intramuscular, haematogenous and aerosol routes. As pigs are often raised in areas of high density, the spread of infection is difficult to control [3–5]. Preventing infection and transmission of the virus is essential in reducing its negative impact on individual farms, and on the entire swine production industry.

It has been reported that as few as 20 virus particles injected intramuscularly into a naïve animal can result in infection [3]. Once an animal is infected, the adverse effects of PRRSV are primarily a consequence of acute viraemia [2]. Therefore, reducing viraemia is important in PRRSV control programmes. In addition, reduced viraemia leads to reduced shedding from infected pigs and thus reduced virus transmission [6,7].

Vaccination is one tool used to prevent PRRSV infection. Vaccination has been shown to mitigate the consequences of infection by reducing clinical signs, viraemia, and lung lesions; all of which contribute to improved health and performance in pigs [6–10].

Various publications have demonstrated that vaccination with PRRS Type 2 modified-live vaccines (MLVs) contribute to successful PRRSV elimination programmes [11]: they improve ADWG [6] and survival rates [6,12] in PRRSV-challenged pigs. Vaccine-derived immunity has also been shown to reduce airborne levels and transmission of circulating viruses [6–8]. This is important in controlling PRRSV in pig-dense and PRRS-prevalent areas.

Increasing genetic diversity of PRRSV threatens herd immunity–posing a challenge to the industry. Therefore, having vaccines that provide heterologous protection is essential [13]. Ingelvac PRRS Type 2 MLV (Boehringer Ingelheim Vetmedica Inc., St. Joseph, Missouri, USA) confers heterologous protection against both Type 1 and Type 2 PRRSV strains [9,12]. The vaccine was a key component in a recent successful elimination project in Denmark [11]. Clinical responses, consequences of infection and vaccination impact an animal’s PRRSV status at various doses of virus.

The objective of this study was to evaluate the dose of virulent PRRSV challenge needed to cause and maintain viraemia in PRRS Type 2 MLV-vaccinated pigs. This study also evaluated vaccinated pigs’ clinical responses and consequences of infection to various doses of virulent challenge. PRRS Type 2 MLV was shown to mitigate the consequences of PRRSV infection at all evaluated PRRSV challenge doses.

## Materials and methods

### Study design and objectives

This was a randomised, blinded vaccination-challenge study involving 95 3-week old pigs. The objective was to evaluate the dosage level of a virulent heterologous PRRSV isolate required to cause and maintain viraemia in vaccinated pigs.

### Animal information

The pigs in this study were born and raised at Wilson Prairie View Farm, WI, USA and owned by Boehringer Ingelheim Vetmedica, Inc., Iowa, USA. To be included in this study, pigs had to be 3 weeks old ±5 days, PRRSV antibody and polymerase chain reaction (PCR) negative, and assessed by the study investigator as being in good health at the start of the study. The pigs originated from 19 different litters and were housed in adequate conditions. All pigs were humanely cared for throughout the study.

### Study procedures

Pigs were randomly assigned to one of five groups (Table 1). On Day 0, 10 pigs in each group were vaccinated with 2 mL PRRS Type 2 MLV intramuscularly, while 10 pigs in Groups 1–4 served as non-vaccinated controls. Group 5 was a non-challenged vaccinated treatment control group. Pigs receiving the same challenge dose were housed together, and vaccinated and non-vaccinated pigs were housed separately.

**Table 1:** Study design for a randomised, blinded, vaccination-challenge study.

Group	PRRS Type 2 MLV vaccinated pigs (n)	Non-vaccinated challenge control pigs (n)	PRRSV challenge dose (log_10_TCID_50_/mL)
**1**	10	10	<1.5
**2**	10	10	2
**3**	10	10	3
**4**	10	10	4
**5**	10	5*	None

*Necropsied on Day 28 (day of challenge)

MLV = modified-live vaccine; n = number; PRRSV = porcine reproductive and respiratory syndrome virus; TCID_50_ = 50% tissue culture infectious dose

On Day 28, all pigs in Groups 1–4 were intranasally challenged with 2 mL virulent heterologous PRRSV challenge isolate (SDSU73; GenBank accession number AY656993 [Boehringer Ingelheim Animal Health]). PRRS Type 2 MLV has 89% similarity to the SDSU73 challenge isolate based on ORF5 sequence.

The concentrations of the challenge isolate ranged from <1.5 to 4 log_10_ 50% tissue culture infectious dose (TCID_50_)/mL (log). Group 5 received no challenge; five pigs from Group 5 received no vaccination to serve as environmental controls. These five environmental controls were necropsied on Day 28 (day of challenge). The 10 remaining pigs in Group 5 continued in the study until study termination (Day 70; Table 1). All pigs in Groups 1–4 were necropsied on Day 70 (42 days after challenge).

#### Determining viraemia and presence of antibodies in serum

Viraemia and PRRSV antibody levels were assessed in serum. Venous whole blood (8–15 mL) was collected on Days 0, 7, 14, 21, 28, 31, 33, 35, 38, 42, and then weekly thereafter until Day 70. To test for viraemia, reverse transcription and real-time PCR (VetMAX PRRSV NA & EU Reagents; Applied Biosystems, Foster City, CA, USA) targeting both Type 1 and Type 2 PRRSV was used. A cycle threshold (CT) cut-off value of 37 was used to determine ‘positive’ or ‘negative’ samples: samples with a CT value of <37 were identified as ‘positive’, and samples with a CT value of >36 were identified as ‘negative’.

Enzyme-linked immunosorbent assay (ELISA [IDEXX PRRS ELISA]) was used to detect PRRSV antibodies in blood serum (Boehringer Ingelheim Health Management Center [Ames, Iowa, USA]) to ensure that all pigs were PRRSV negative before the study started and that seroconversion had occurred following vaccination. Results were recorded as a sample-to-positive (S:P) ratio, and values ≥0.4 were considered PRRSV positive.

#### Monitoring biological effects

To determine the presence of pyrexia (defined as a rectal temperature >40 °C), rectal temperatures were collected on Day 27 (1 day before challenge) and daily thereafter until Day 42. To assess ADWG, pigs were weighed at study start, before challenge on Day 28, and on Day 70 (day of study termination).

All pigs were observed daily from Day 28 (before challenge) until Day 70 for clinical signs of disease associated with PRRSV infection. The following clinical signs were monitored: respiratory problems, abnormal behaviour and cough. Clinical signs were recorded as 1 (present) or 0 (absent).

#### Statistical methods

Animal randomisation and statistical analysis were conducted by Boehringer Ingelheim Animal Health (SAS version 9.4). Randomisation was conducted using an incomplete block design where littermates were randomised to vaccinated and non-vaccinated groups within each challenge dose group.

ADWG and number of days pyrexic were analysed using a linear mixed model with random effects (room, pen within room) and a residual error term. Fixed effect terms included in the initial model were group-specific intercept and slope terms representing a linear regression model, with challenge dose (log_10_ scale) as the independent variable. The model was simplified to include only statistically significant terms. Vaccine and challenge control groups were then statistically compared either between groups (common slope for both groups) or between groups at specific challenge dose values (unique slope for each group). A P-value <.05 was used to indicate statistical significance.

### Animal care and welfare

Pigs were housed at the animal facilities at Veterinary Resources Inc., Cambridge, Iowa, USA, for the duration of the study, in raised plastic tubs with plastic slatted flooring. Pen dimensions were 5 ft x 4 ft with five pigs per pen. Pigs were fed a commercial ration containing antibiotics that was made available *ad libitum* via nipple waterer. Floor and feeder space met requirements set forth in the 2010 Consortium ‘Guide for the Care and Use of Agricultural Animals in Agricultural Research and Teaching’ [14]. Euthanasia was performed by overdose intravenous injection of barbiturates (390 mg/mL of pentobarbital sodium and 50 mg/mL phenytoin sodium) at a dosage of 5 mL/100 lb body weight. The study was reviewed and approved by the local Institutional Animal Care and Use Committee, AMVC WeSearch DBA VRI.

## Results

### Viraemia

All pigs except six were found to have seroconverted following vaccination. These six pigs were omitted from the analysis: two vaccinated pigs challenged with 4 log PRRSV (Group 4) and four vaccinated pigs challenged with 3 log PRRSV (Group 3). Day 31 (3 days after challenge), all pigs had become viraemic (n = 84).

**Fig 1:**
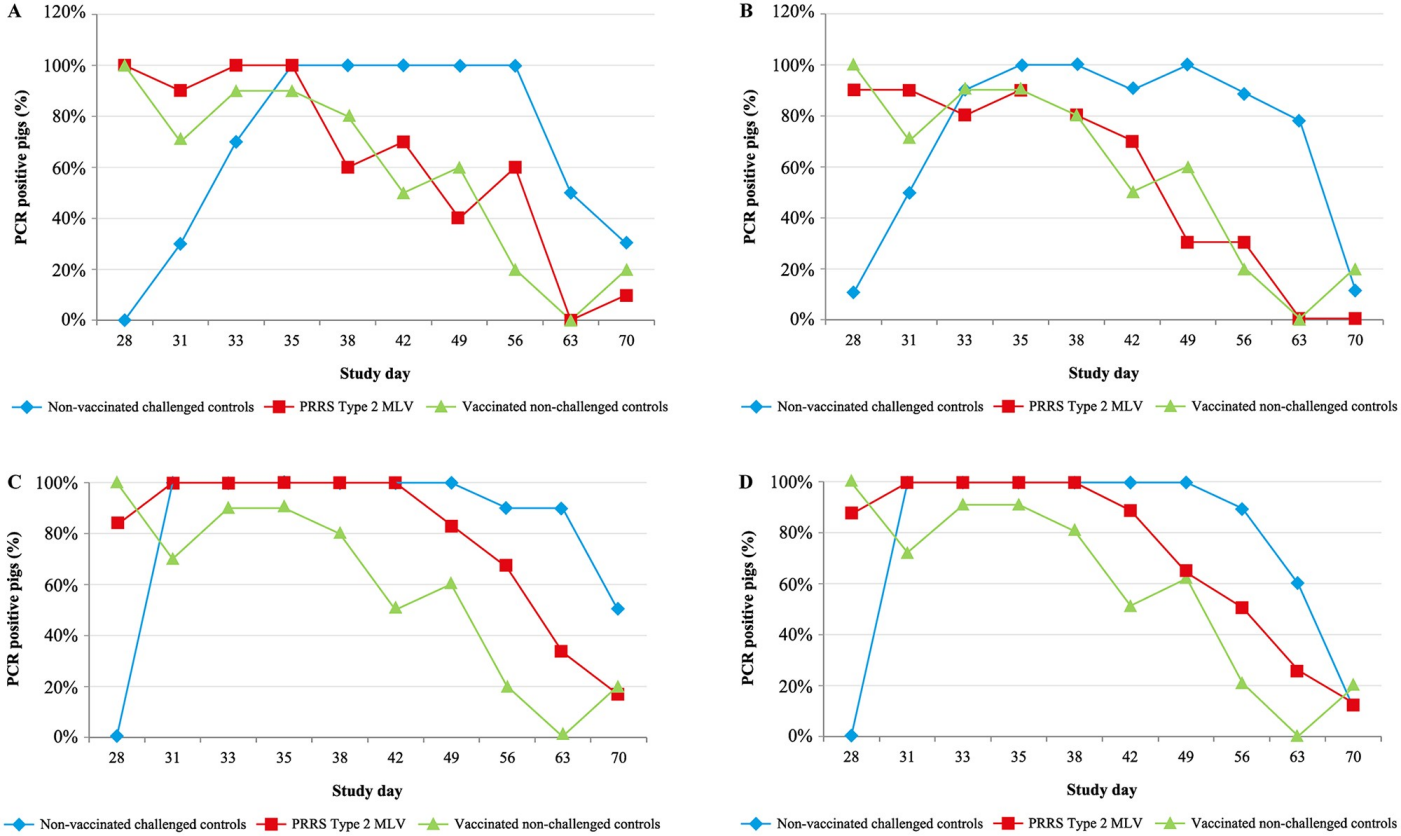
Viraemia in pigs challenged with PRRSV. a) Percentage of PCR positive pigs challenged with <1.5 log virulent PRRSV strain. b) Percentage of PCR positive pigs challenged with 2 log virulent PRRSV strain. c) Percentage of PCR positive pigs challenged with 3 log virulent PRRSV strain. d) Percentage of PCR positive pigs challenged with 4 log virulent PRRSV strain. MLV = modified-live virus; PCR = polymerase chain reaction; PRRS = porcine reproductive and respiratory syndrome; PRRSV = porcine reproductive and respiratory syndrome virus; TCID_50_ = 50% tissue culture infectious dose.

The percentage of PCR positive pigs started decreasing in vaccinated groups challenged with 4 log (Group 4) between Days 38 and 42, and in vaccinated groups challenged with 3 log (Group 3) between Days 42 and 49. Reduction in viraemia occurred earlier (between Days 35 and 38 [7–10 days post-challenge]) in vaccinated pigs challenged with <1.5 and 2 log PRRSV (Groups 1 and 2, respectively), and showed a similar trend to vaccinated, non-challenged pigs (Fig 1).

In all non-vaccinated, challenged groups, the percentage of PCR positive pigs remained higher for longer compared with the corresponding vaccinated group (Fig 1).

As the challenge dose decreased, the percentage of viraemic pigs in the vaccinated groups also decreased over time throughout the challenge phase. In the non-vaccinated groups, pigs had similar post-challenge viraemia profiles regardless of the challenge dose they received and higher percentage of PCR positives compared to vaccinated treatment groups during the post-challenge phase of study.

### Pyrexia

Vaccinated groups had significantly fewer average pyrexic days than non-vaccinated groups at all levels of challenge (P <.05). Table 2 shows estimated number of days pyrexic for challenge doses adjusted to 1, 2, 3 and 4 log based on model prediction. Estimations show that vaccinated pigs challenged with 1 log and 2 log PRRSV would show no significant difference in average days pyrexic post-challenge compared with vaccinated, non-challenged pigs (Table 2).

**Table 2:** Mean number of days vaccinated, challenged pigs were pyrexic compared with non-vaccinated pigs post-challenge.

Treatment Group	PRRSV challenge dose (log_10_TCID_50_/mL)	Estimated mean number of days pyrexic				
		No challenge	1	2	3	4
PRRS Type 2 MLV		1.8	1.4*	1.0*	4.2*	4.4*
Non-vaccinated challenge control		-	6.0	10.0	8.8	11.2

*Statistically significant difference (P <.05) in number days pyrexic between Type 2 PRRS. MLV and challenge control groups based on model prediction.

MLV = modified-live vaccine; PRRS = porcine reproductive and respiratory syndrome; PRRSV = porcine reproductive and respiratory syndrome virus; TCID_50_ = 50% tissue culture infectious dose

**Fig 2:**
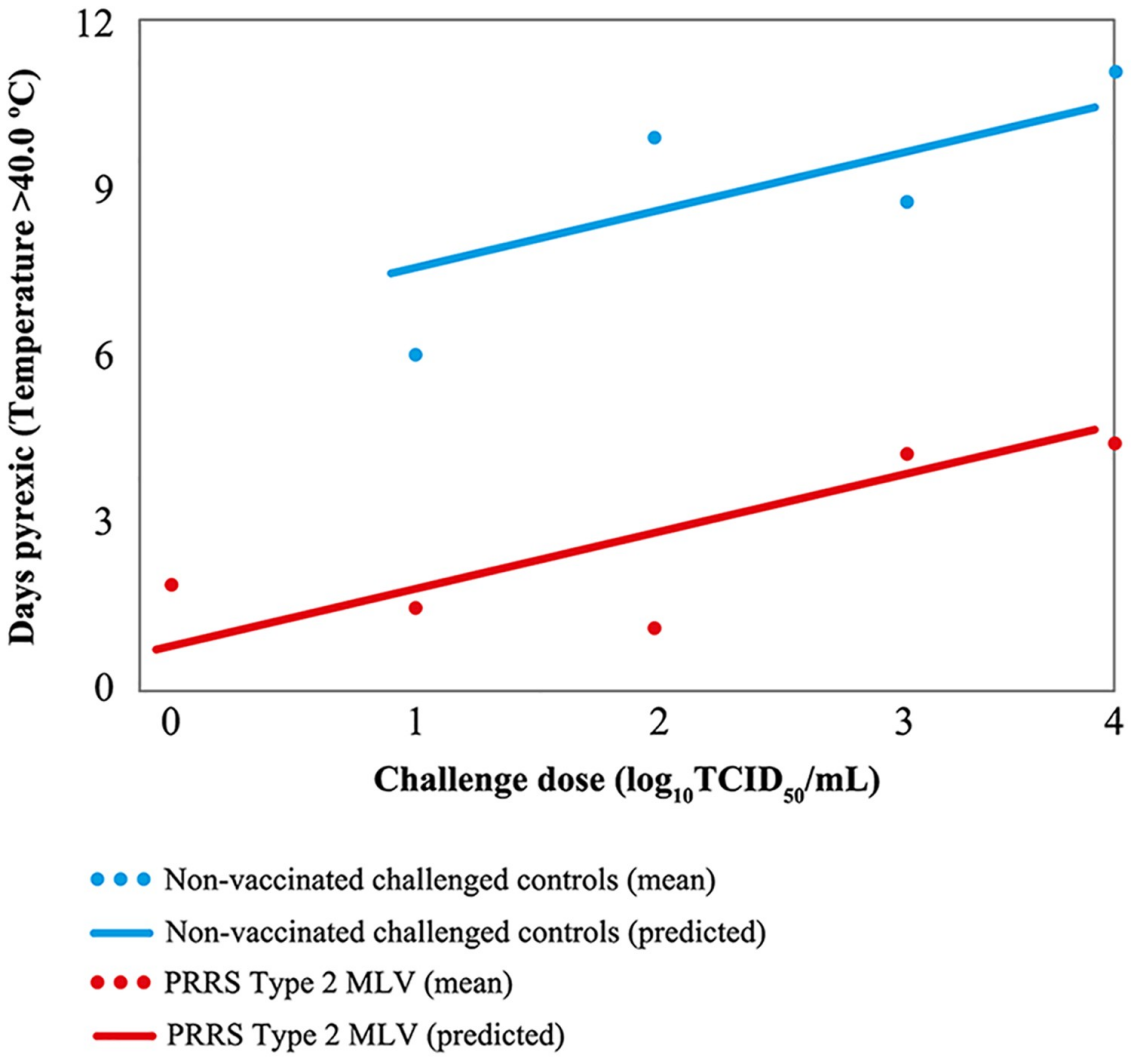
Estimated number of days pyrexic by challenge dose based on model prediction. Regression intercept and slope utilised to predict number of days pyrexic. Individual data points show mean average number of days pyrexic for each challenge dose group as recorded between Days 28 and 70. MLV = modified-live virus; PRRS = porcine reproductive and respiratory syndrome; TCID_50_ = 50% tissue culture infectious dose.

Fig 2 shows the estimated number of days pyrexic for challenge doses 1, 2, 3 and 4 logs based on model prediction. As challenge dose increased, the number of days with pyrexia increased among both vaccinated and non-vaccinated groups.

### Average daily weight gain (ADWG)

ADWG was greater for vaccinated, challenged pigs compared with non-vaccinated, challenged pigs in all challenge dose groups. ADWG was significantly greater in groups challenged with <1.5, 2 and 3 log PRRSV (Groups 1, 2 and 3, respectively [P <.05]). ADWG for vaccinated pigs challenged with <1.5 and 2 log PRRSV (Groups 1 and 2, respectively) had an ADWG of 0.74 and 0.77 kg/day respectively, which is numerically similar to that of vaccinated, non-challenged pigs (0.76 kg/day [Table 3]).

**Table 3:** ADWG by treatment group and challenge dose.

Treatment Group	PRRSV Challenge Dose (log_10_TCID_50_/mL)	ADWG (kg/day)				
		No challenge	<1.5	2	3	4
PRRS Type 2 MLV		0.76	0.74*	0.77*	0.59*	0.64
Non-vaccinated challenge control		-	0.56	0.52	0.48	0.54

*Statistically significant difference (P <.05) in ADWG between Type 2 PRRS MLV and challenge control groups based on model prediction.

ADWG = average daily weight gain; MLV = modified-live virus; PRRS = porcine reproductive and respiratory syndrome; PRRSV = porcine reproductive and respiratory syndrome virus; TCID_50_ = 50% tissue culture infectious dose.

**Fig 3:**
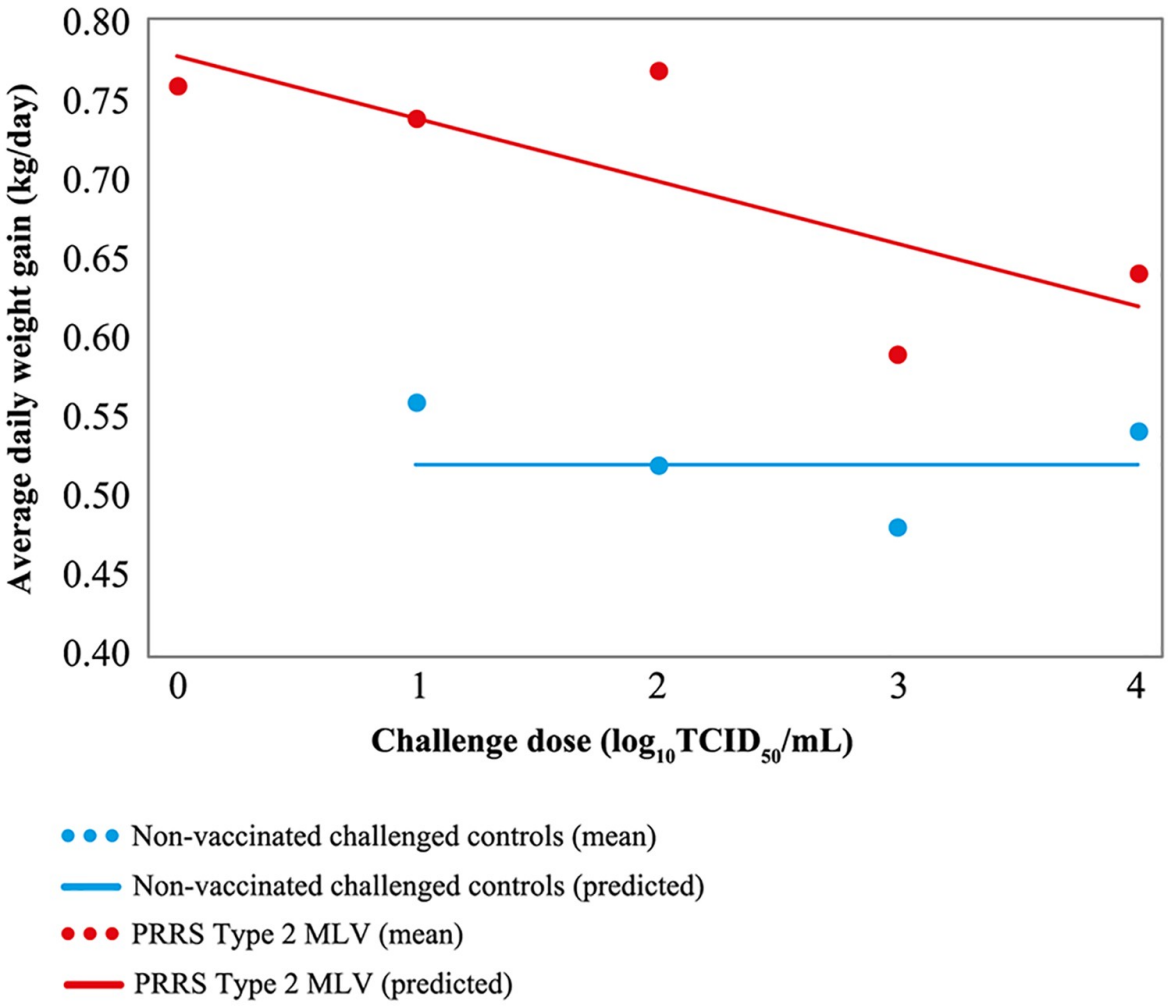
Estimated ADWG by challenge dose based on model prediction. Regression intercept and slope utilised to predict ADWG. Individual data points show mean ADWG for each challenge dose group as recorded between Days 28 and 70. ADWG = average daily weight gain; MLV = modified-live virus; PRRS = porcine reproductive and respiratory syndrome; TCID_50_ = 50% tissue culture infectious dose.

Fig 3 shows estimated ADWG for challenge doses 1, 2, 3 and 4 log based on model prediction. There was a significant increase (P <.05) in the ADWG based on the challenge dose in the vaccinated groups: ADWG increased 0.039 kg/day for each 1 log decrease in challenge dose. In all non-vaccinated challenged groups, ADWG was reduced, with no significant difference across all challenge doses.

### Clinical signs

Pigs were observed daily from Day 28 (before challenge) until Day 70 for clinical signs of disease associated with PRRSV infection. Pigs were monitored for respiratory problems, abnormal behaviour and cough. Vaccinated, challenged groups experienced a lower mean and median duration of clinical signs compared with non-vaccinated, challenged groups at all challenge doses (Table 4). Vaccinated groups challenged with <1.5 log and 2 log had the same mean and median duration of clinical signs as non-challenged, vaccinated groups.

**Table 4:** Average number of days pigs showed any clinical signs, by treatment group and challenge dose.

Treatment group		PRRSV challenge dose (log_10_TCID_50_/mL)	Average number of days of clinical signs*				
			No challenge	<1.5^†^	2	3	4
PRRS Type 2 MLV	n		10	10	10	6	8
	Mean		0.2	0.2	0.3	1.3	1.1
	SD		0.6	0.4	0.5	2.0	2.1
Non-vaccinated control	n		-	10	10	10	10
	Mean		-	3.8	7.2	7.0	8.7
	SD		-	5.6	4.8	6.5	4.1

*Any clinical signs from day of challenge to day of necropsy.

^†^Below assay limit of detection.

MLV = modified-live vaccine; n = number of pigs; PRRS = porcine reproductive respiratory virus; SD = standard deviation; TCID_50_ = 50% tissue culture infectious dose.

On Day 52, one pig from Group 1 was found dead. After necropsy, the pig was diagnosed with severe pneumonia and associated pleuritis. There were no other deaths in this study.

## Discussion

The objective of this study was to evaluate the dose of virulent PRRSV challenge needed to cause and maintain viraemia in PRRS Type 2 MLV-vaccinated pigs. This study also evaluated vaccinated pigs’ biological and clinical responses to various doses of virulent challenge. The study results show that vaccination with PRRS Type 2 MLV reduces viraemia following challenge at all concentrations of heterologous PRRSV challenge compared with non-vaccinated pigs. At lower challenge doses (<1.5 and 2 log), PRRS Type 2 MLV-vaccinated pigs performed similarly to non-challenged pigs.

The longest period of detectable viraemia observed among the vaccinated groups was in the two high-dose challenged groups (3 and 4 log). The peak post-challenge viraemia in these groups remained for longer than the peak viraemia observed in the low-dose challenged groups (<1.5 and 2 log). These low-dose challenged, vaccinated pigs had similar levels of viraemia as non-challenged, vaccinated control pigs. This suggests that vaccinating pigs with PRRS Type 2 MLV helps prevent a rise in viraemia levels at low doses of PRRSV challenge.

For all groups that were challenged, vaccinated pigs began recovering from PRRSV before their non-vaccinated counterparts. The percentage of PCR positive pigs in each vaccinated challenge dose group began decreasing between Day 38 (10 days post-challenge) and Day 49 (14 days post-challenge). Vaccination accelerated recovery in pigs at all challenge doses; mean viraemia levels began decreasing in vaccinated pigs before non-vaccinated pigs at all challenge doses.

Vaccination with PRRS Type 2 MLV also reduced the incidence of clinical signs that are common in PRRSV infections. Vaccination reduced pyrexia and mitigated clinical signs leading to improved ADWG. These findings suggest that PRRS Type 2 MLV offers a level of heterologous protection against the consequences of PRRSV infection at all challenge doses.

ADWG is a primary economic driver of health and performance in swine production [1]. Reduced ADWG results from loss of appetite and reduced feed intake; usually caused by high temperatures and pneumonia (with associated respiratory problems) as a consequence of PRRSV infection [2]. Reducing the impact of PRRSV infection on ADWG is key in improving the profitability of swine production [1].

The current study is the first to use a challenge-dose model to evaluate the effect of PRRSV challenge in vaccinated animals compared with non-vaccinated animals. The study was valid as all pigs were antibody and PCR negative for PRRSV prior to study start, and the majority became viraemic following challenge. The lack of seroconversion of a small number of pigs was likely caused by variation in host vaccination response, and this is a common observation in vaccination studies. However, there are limitations to this study. Firstly, the sample sizes were small and so the findings need to be confirmed in larger studies. Secondly, repeating this study using pigs of different ages and immune status will help us discover whether our conclusions apply equally to all pigs, rather than only naïve pigs. Thirdly, the current study shows that PRRS Type 2 MLV protects against the SDSU73 challenge isolate; therefore, we would need to conduct further studies to confirm that these results are repeatable against a different PRRSV challenge isolates. However, previous heterologous efficacy studies have shown that PRRS Type 2 MLV is effective against several relevant PRRS field virus challenges; vaccinated pigs had significantly reduced lung lesions, post-challenge viraemia, clinical signs and improved ADWG compared with non-vaccinated challenged animals [6,10,15]. Based on the results of other heterologous efficacy studies, one might suggest that this model would give similar results with other heterologous challenge isolates, and warrants further investigation. A final limitation is that, as with all controlled experiments, it is unknown how these results would translate to field situations. Additional efforts to apply the learnings from this study in relevant field models may be warranted. In the future, this challenge-dose model could be used to address whether a particular vaccination protocol is superior to other vaccination protocols in protecting PRRSV infected pigs at a range of challenge doses. This study demonstrates that vaccinated pigs exposed to moderate to low levels of virus are just as healthy as pigs that were not challenged at all. These findings support efforts such as regional control and system-based PRRSV control programmes that focus on protocols for maintaining immunity and minimising exposure [11,16]. This study also shows that the consequences of infection were significant in non-vaccinated pigs regardless of challenge dose. This supports other studies that concluded that non-vaccinated, PRRSV-naïve pigs can become infected by low doses of PRRSV [3]. Where there is a risk of PRRSV infection, vaccine-derived immunity plays an important role in mitigating the consequences, and improving health and performance. Therefore, the ability of PRRS Type 2 MLV to protect against the consequences of PRRSV infection in young pigs makes PRRS Type 2 MLV an important component of PRRSV control projects.

## Conclusions

- PRRS Type 2 MLV protects against a wide range of virulent PRRSV challenge doses tested.
- Vaccine-derived immunity can mitigate the consequences of infection by reducing post-challenge viremia, reducing clinical signs, improving health and ADWG performance. For non-vaccinated pigs, the impact on viraemia and ADWG was similar at all levels of challenge. Where there is any risk of PRRSV exposure, this data supports the use and benefit of vaccination.
- Maximising ADWG in young pigs is key to maximising the profitability of swine production. PRRS Type 2 MLV protects against the negative impact of PRRSV on ADWG, and helps improve the profitability of swine production systems.
- This study shows that lower levels of challenge have minimal impact on health and performance of vaccinated pigs, as they performed similar to pigs that were not challenged–supporting PRRSV vaccination control programmes targeted at maintaining immunity and minimising exposure.

## Supporting information

S1 Dataset Raw data from clinical investigations. (XLSX)
